# Multi-modal triggered-release sonodynamic/chemo/phototherapy synergistic nanocarriers for the treatment of colon cancer

**DOI:** 10.3389/fbioe.2024.1439883

**Published:** 2024-07-22

**Authors:** Yun Zhou, Yueyang Gao, Nannan Yao, Guozhi Lu, Chuyu Dong, Kexin Wang, Junfeng Zhang, Jing Sun, Ke Li, Xueping Li

**Affiliations:** ^1^ College of Clinical Medicine, Xi’an Medical University, Xi’an, China; ^2^ The Second College of Clinical Medicine, Xi’an Medical University, Xi’an, China; ^3^ Xi’an Key Laboratory for Prevention and Treatment of Common Aging Diseases, Translational and Research Centre for Prevention and Therapy of Chronic Disease, Institute of Basic and Translational Medicine, Xi’an Medical University, Xi’an, China; ^4^ College of Medical Technology, Xi’an Medical University, Xi’an, China

**Keywords:** colon cancer, phototherapy, sonodynamic therapy, chemotherapy, muti-modal release, synergistic effect

## Abstract

Most colon cancer patients are diagnosed at an advanced stage, with a grim prognosis. In clinical, various combination therapies have been employed to enhance the efficacy of colon cancer treatment. The essence of combined treatment is the judicious selection and combination of various treatment units. Phototherapy (PT), sonodynamic therapy (SDT), and chemotherapy are treatment modalities that rely on the active molecules to treat tumors, and have been demonstrated to synergistically enhance tumor treatment efficacy. However, the differences in the metabolism of active molecules and hypoxic microenvironment of tumors have limited the synergistic effects of the aforementioned methods. To address this significant issue, in this study, we utilized polydopamine (PDA) as the encapsulated material to form a rigid shell that contains the therapeutic molecules IR-780 and methotrexate (MTX) on the surface of perfluorohexane (PFH) microdroplets through self-assembling method to develop an SDT/chemotherapy/PT combined nanoparticles (SCP NPs). Transmission electron microscopy (TEM) revealed that the nanoparticles exhibited a hollow shell structure, with an average size of approximately 100 nm. SCP NPs have excellent stability and biocompatibility in both *in vitro* and *in vivo*. The absorption and emission spectrum of the loaded IR-780 did not exhibit any significant shift, and the photothermal temperature rose to 92°C. Their ultrasonic cavitation effect was good and their cell inhibitory effect of MTX was maintained. SCP NPs can achieve multi-modal triggered release through ultrasound, laser irradiation, and pH, ensuring a simultaneous accumulation of therapeutic molecules in the tumor area and effectively alleviating tumor hypoxia. Additionally, both the near-infrared fluorescence (NIF) signal and the ultrasonic cavitation signal of the nanoparticles can be utilized for tracking and monitoring treatment efficacy. Most notably, SCP NPs exhibited outstanding synergistic treatment effects at low intervention levels, resulting in a 67% cure rate of tumors. These results provide an experimental basis for developing the new clinical treatments for colon cancer.

## 1 Introduction

Colon cancer is a highly aggressive malignant tumor. It is the third most prevalent cancer and the second leading cause of death from cancer in the world, and the number of cases is increasing rapidly year by year. In 2023, more than 150,000 people worldwide were diagnosed with colon cancer, and over 50,000 patients died from this disease. (Siegel et al., 2023). Colon cancer has no obvious early symptoms and is prone to metastasize. Most patients are already in the late stage when diagnosed, with poor prognosis and high mortality rate (Favoriti et al., 2016). In current clinical practice, it is accepted that two or more combined therapies can improve the efficiency of tumor treatment (Yap et al., 2013; Mokhtari et al., 2017). Due to the complexity, diversity, and heterogeneity of tumors, the synergetic effect between different treatments is poor, reducing the therapeutic effect and causing mutual antagonism of the combined treatment methods. Therefore, reasonable selecting and matching of treatments are the core of combination therapy. A synergetic effect with high response efficiency and few by-products can be introduced into combination therapy to minimize its side effects and amplify its therapeutic effects.

At present, many new tumor treatment methods, such as immunotherapy (Xi et al., 2020; Xiao et al., 2021), photothermal therapy (PTT) (Jung et al., 2018; Zhang et al., 2019; Xu et al., 2021), photodynamic therapy (PDT) (Wang et al., 2017; Li et al., 2021a; Yang et al., 2021) and chemodynamic therapy (Ren et al., 2020; Tang et al., 2022; Zhao et al., 2023), have been developed. Many researchers used PTT and PDT methods to conduct research on colon cancer treatment (Goodrich et al., 2010; Azhdarzadeh et al., 2016; Cabeza et al., 2020; Chen et al., 2020; Freitas et al., 2023; Rodrigues and Correia, 2023). The photosensitizer is the essence of PTT and PDT. However, shortcomings of traditional photosensitizers such as the tendency to aggregate under physiological conditions, insufficient accumulation in tumor sites, and insufficient light penetration limited the application of phototherapy (PT) in eradicating tumors. Later, some researchers developed sonodynamic therapy (SDT) based on PDT. This method relies on the cavitation effect of vacuolating agents induced by ultrasound (US) and sound sensitizers (SSs) to produce highly cytotoxic reactive oxygen species (ROS) for triggering the apoptosis of tumor cells. Many studies have focused on the treatment of in-depth tumors by SDT (Gao et al., 2013; Li et al., 2019; Liang K. C. et al., 2020). SSs are mainly divided into two types: organic and inorganic SSs. Organic SSs are mainly developed from photosensitizers. Compared with inorganic SSs, organic SSs have the advantages of easy modification, *in vivo* degradability, and large ROS production. However, organic SSs are usually hydrophobic and are easily accumulated and cleared *in vivo*, showing certain side effects (Hu et al., 2014; Choi and Kim, 2021). US can penetrate soft tissue and achieve energy deposition in deep lesion areas (Zhang et al., 2021; Li R. T. et al., 2022). According to the frequency, ultrasound treatment methods can be categorized into high intensity focused ultrasound (HIFU) and low intensity ultrasound (LUS). LUS has a longer wavelength and lower energy than HIFU does. The heat generated by LUS is not enough to directly ablate tumor cells, but LUS is safer for the body, and SDT mainly relies on LUS (Chen et al., 2023). In addition to acting on tumor cells, LUS can also be used for the delivery and release of anti-tumor drugs through triggering the carriers that transport drugs or changing the permeability of tumor cell biofilms to make it easier for drugs to enter target cells and achieve better therapeutic effects (Awad et al., 2021; Canavese et al., 2018). Tumor tissues usually have a hypoxic microenvironment, which causes tumor cells to adaptively change their metabolism and survival strategies and reduce their sensitivity to oxidative stress, causing an obvious resistance of tumors to a variety of ROS-dependent treatments including PDT and SDT. In addition, hypoxia makes tumor cells more likely to stay in the G1/G0 phase rather than entering the S phase, reducing the effectiveness of treatments. Perfluorocarbon compounds are LUS enhancers and FDA-approved high oxygen-carrying materials (Luo et al., 2007). This feature provides foundation for tumor hypoxia mitigation and ROS-related tumor treatments. Both ROS and hyperthermia can cause DNA damage and trigger cell apoptosis. Methotrexate (MTX) is an inhibitor of DNA synthesis and can induce cell apoptosis. This shows that the compound has complementarity with PTT, PDT and SDT in mechanisms and can be used for synergistic treatment. Therefore, another core issue becomes how the effective integration of multi-functional modules is achieved.

The development of nanomaterials and technologies has promoted the advancement of multifunctional diagnostic and therapeutic platforms. Those platforms can effectively integrate different functional molecules on a nanoscale particle and be applied for tumor targeting, providing important technical support for combined tumor treatment strategies. The essence of establishing such integrated platform is the functional materials. Firstly, the selected materials must have good biocompatibility. Secondly, they must be able to directly and effectively assemble with various functional molecules. Finally, the preparation process must be simple and robust. Polydopamine (PDA), as a synthetic analog of natural melanin, has many biological functions, including broad optical absorption, metal ion chelation, free radical scavenging, antioxidant activity, and neuroprotective effects. These properties make PDA stand out among many polymer nanomaterials in the biomedical field (Král et al., 2023; Chinchulkar et al., 2022). In addition, functional groups such as catechol, amines, and imines are rich in the aromatic ring structure of PDA. Therefore, PDA can also load drug molecules through π-π interactions, and the reaction only requires simple and mild conditions. PDA has been widely used in drug delivery systems (Jabbar et al., 2023). Its abundant catechol groups allow molecular reactions with thiols or amino groups through Michael addition or Schiff base reactions, promoting the surface functionalized adhesion of PDA with various nanomaterials and biomolecules. PDA has been layered on the surface of various nanomaterials (Lee et al., 2007).

**SCHEME 1:**
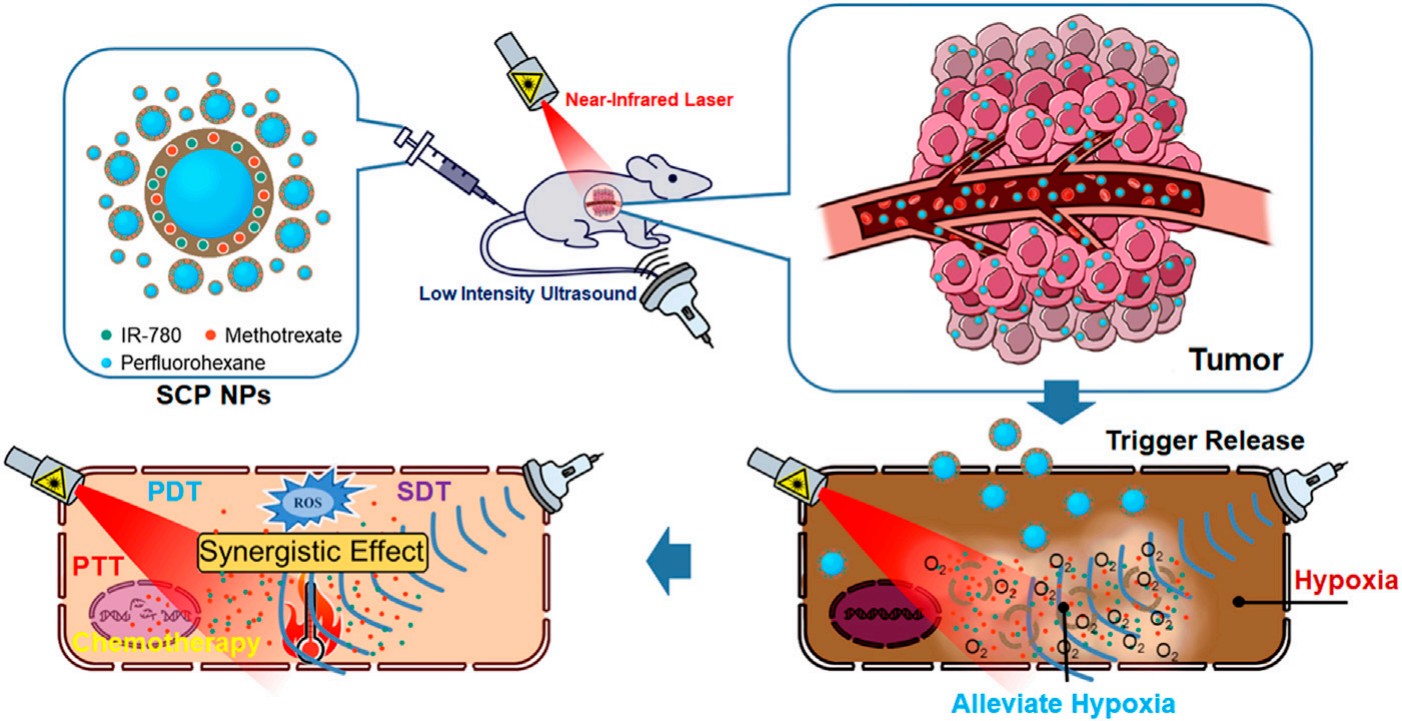
Schematic diagram of the delivery, release, and treatment process of SCP NPs.

Polymer hollow structures have attracted widespread attention due to their low density, high specific surface area, excellent surface permeability, large loading capacity, and great morphology controllability (De Cock et al., 2010). PDA can spontaneously deposit on various material surfaces. The combination of PDA and core templates represents a versatile preparation strategy for hollow nanostructures. In this strategy, researchers use hard particles such as polymer colloids, or soft particles such as emulsions and droplets as templates, and coats a PDA layer on the template surfaces to achieve a capsule-like shelling (Zhang et al., 2011; Wang et al., 2015; Xu et al., 2011; Yeroslavsky et al., 2013). Based on the original work of our team (Zhu et al., 2019; Li et al., 2021b), in this study, we controlled the hybrid assembly process of PDA and therapeutic molecules through stabilizers and redox auxiliaries (Bernsmann et al., 2011). As a therapeutic molecule, indocyanine dyes have been widely used in fluorescence imaging and PDT. Among the indocyanine dyes, IR-780 has a clear SDT effect (Ren et al., 2021; Guan et al., 2023; Chen et al., 2024). We loaded IR-780 and MTX, a chemotherapy drug that can interfere with tumor cell metabolism and proliferation, on the surface of perfluorohexane (PFH) nanoemulsion droplets to form a PDA functionalized shell with a certain rigidity. Through a series of evaluation tests, we have developed a multifunctional diagnosis and treatment nano-platform, providing a basis for new clinical treatment methods of colon cancer. The entire research concept and process are depicted in Scheme 1. The nanoparticles consist of an external rigid shell with internal liquid droplets. Administration is done via intravenous injection, followed by ultrasound-triggered fragmentation at the tumor site to release PFH and O_2_, alleviating tissue hypoxia. Ultimately, synergistic therapeutic effects are achieved through multiple combined interventions.

## 2 Materials and methods

### 2.1 Materials

Dopamine hydrochloride, IR-780, MTX, PFH, coumarin-6, and doxorubicin (DOX) were purchased from Shanghai Macklin Ltd. FS-63 was purchased from Guangzhou Ltd. DMEM high-glucose medium and trypsin were products of Cytiva Hyclone PLC. Dihydroethidium (DHE) ROS fluorescent probe was purchased from Beijing Solarbio Ltd. Cell culture antibiotics, CCK-8, DAPI kit, hypoxia inducible factor-1α (HIF-1α) antibody, and intracellular ROS detection kit were purchased from Shanghai Beyotime Ltd. The other reagents were purchased from Sinopharm Corp. The cell lines, include HUVEC, IMR-90, HL-7702, SW48, CT-26-luc (luciferase reporter gene marker) were derived from ATCC and purchased from Shanghai Coweldgen scientific Ltd. All experimental animals were purchased from Beijing HFK Bio PLC.

### 2.2 The synthesis of SCP NPs

The preparation process of SCP NPs was developed by referring to the process of Zhu and Li et al. (Zhu et al., 2019; Li et al., 2021a). 100 μL PFH and 120 μL FS-63 were mixed and dispersed into 3 mL Tris solution (pH = 9.0) under high-speed stirring. Then, 100 μL of a mixed solution containing IR-780 (at a concentration of 6 mg/mL) and MTX (at a concentration of 10 mg/mL) in DMSO, and 100 μL of FS-63 were added to the dispersion. The dispersion was treated with ultrasound (150 W) for 5 min. Finally, 100 μL of 3% H_2_O_2_ and 20 mg of dopamine hydrochloride were added to the dispersion. The reaction container was sealed and rotated in the dark for 48 h, followed by dialysis to remove DMSO and other soluble impurities to obtain SCP NPs. SCP NPs were primarily tested by a Malvern nano sizer (Nano-90s, Malvern, United Kingdom).

### 2.3 The characterization of SCP NPs

The morphology of SCP NPs was observed with a transmission electron microscopy (TEM, Tecnai G2 F20, FEI, US). The water was removed from the nanoparticles by a freeze dryer (LGJ-12, Songyuan, Beijing, China). The sample was soaked in acidic DMSO (pH = 4.0) to extract IR-780 and MTX, and the encapsulation rate and drug loading capacity were measured, respectively. The absorption spectra of the samples were measured with a UV-visible spectrophotometer (T6, Persee, Shanghai, China). The fluorescence emission spectrum of IR-780 in the sample was measured using a fluorescence photometer (F98, SAID, Shanghai, China). The concentration of IR-780 in the sample within 72 h was tested by dialysis method to determine the release degree. In terms of photothermal conversion, an 808 nm laser (BOT808-5W-GQ, BOT, Xi’an, China) was used to irradiate the sample, and a thermal imager (EX6, FILR, US) was used to monitor the temperature rise. The photothermal test contents include stability, concentration, power, components, and dispersion solutions. And then, the adipose and muscle tissues were used to cover the sample solution surface for an *in vitro* temperature rising test with block. The subcutaneous and intramuscular heating test of the sample was conducted in BALB/c mice. The ultrasonic triggering of SCP NPs was tested with an ultrasonic imaging system (voluson E8, GE, United States). The control group was the medical ultrasonic cavitating agent, SonoVue. SCP NPs were sonicated by a Doppler ultrasonic instrument (DC-25, Mindray, Shenzhen, China), and the IR-780 fluorescence signal in the sample was detected with a *In Vivo* Imaging System (Visque *In Vivo* Smart-LF, Korea). The release rate of SCP NPs was calculated. SCP NPs were treated overnight with solutions with different pH, and the IR-780 concentration was tested to calculate the release rate. The foaming of the sample during the heating process was observed, and the release rate of IR-780 was calculated based on the fluorescence intensity.

### 2.4 Cytotoxicity and *in vitro* combined intervention effect testing of SCP NPs

The cytotoxicity of the sample and its main components was tested by the CCK-8 assay. The cells lines included normal cell lines (HUVEC, IMR-90, HL-7702) and colon cancer cell lines (CT-26-luc and SW48). Cells that had grown to the logarithmic phase were digested with trypsin to prepare a cell suspension. After the concentration was calculated, the cells were planted in a 96-well plate (8,000–12,000 cells/well, 37°C, 5% CO_2_). After the cells adhere, the sample was added to each well according to the concentration gradient. When the cells in the control wells grew to 90%, the medium was replaced with colorless medium containing CCK-8 (10%) and they were incubated for 1–1.5 h. A microplate reader (TACAN, Spark, US) was used to read the plate at 450 nm and cell viability was calculated. The combined intervention effect of SCP NPs was evaluated through CCK-8, live and dead cell staining, colony formation, and ROS production. The cell lines were CT-26-luc and SW48. The CCK-8 method was performed as described above, except that the number of cells per well was 20,000. After adding the sample, the cells were irradiated with an 808 nm laser, and then were incubated for 12 h. The cell survival rate was calculated in the same way. In the live and dead cell staining test, 60,000 cells were seeded in each well of a 6-well plate. After grew to 60%–70%, the cells were divided into control, MTX, IR-780, and SCP NPs treated groups. The IR-780 and SCP NPs groups were sonicated for 10 min before laser irradiation. The irradiation conditions were consistent with those of the CCK-8 test. Afterwards, the cells were incubated for 12 h, stained with a live-dead cell staining kit, and observed with an inverted fluorescence microscope (DMi8, Leica, Germany). In the colony formation experiment, the cell suspension was placed into 1.5 mL centrifuge tubes at 2,000 cells/mL, which was then grouped in the same way as live-dead cell staining test. The cell suspension was then moved to a culture dish. After the number of cells exceeded 50, colonies were stained and observed. ROS is an important detection indicator in PT and SDT. The cells were planted in a 6-well plate at 40,000 cells/well. After adhered to the wall and grew normally, the cells were divided into groups and given corresponding samples and interventions. After the intervention, the cells were incubated for 6 h, and the ROS kit was used to stain the cells. The fluorescence signals of the cells were observed through a fluorescence microscope.

### 2.5 Cellular internalization and affinity testing of SCP NPs

In order to effectively observe the internalization effect of nanoparticles, SCP NPs were fluorescently labeled. The fluorescent dyes were coumarin-6 (green) and DOX (red). SW48 cells were planted in confocal dishes at 20,000 cells/dish. After culturing for 24 h, fluorescently labeled SCP NPs were added and cell samples were collected at different time points. The cells were fixed with paraformaldehyde, and the nuclei were stained with DAPI. Cell internalization was observed with a laser confocal microscopy (FV3000, Olympus, Japan). The cells were pre-treated with an active endocytosis inhibitor, NaN_3_ (concentration 1 mg/mL), and then fluorescence labeled SCP NPs were added. The optimal incubation time was determined based on the internalization results. The cells were fixed with paraformaldehyde, and the nuclei were stained with DAPI. Confocal microscopy was used to detect intracellular fluorescence levels and perform quantitative analysis of fluorescence intensity.

### 2.6 Biocompatibility testing of SCP NPs

Since SCP NPs need to be administered intravenously, their impact on red blood cells were evaluated through hemolysis experiments. A 2% red blood cell suspension was prepared. After grouping, SCP NPs, IR-780, PFH, FS-63, and MTX were added, respectively. Pure water, physiological saline, and 0.1% Triton X-100 were selected as the control groups. The cells and samples were incubated at 37°C for 3 h. The suspension was then centrifuged at 8,000 rpm for 10 min and the precipitation was observed. After discarding the supernatant, an equal amount of pure water was added to each tube. The suspension was incubated at 37°C for 4 h. The absorption wavelength of 540 nm for each sample was measured using a microplate reader, and the hemolysis rate was calculated.

In the acute toxicity experiment, 40 BALB/c mice, half male and half female, weighing approximately 20 g, were randomly divided into 4 groups. SCP NPs, PFH + FS63, and IR780 were injected intravenously, respectively, and the control group was injected with physiological saline. The injection dose was the maximum concentration of SCP NPs, and the concentration of the corresponding components was equivalent to the concentration of SCP NPs. Fasted for 6 h before administration, and food and water were restored 2 h after injection. The mice were observed continuously for 14 days. The physical signs and body weight were recorded, and the survival rate of mice was calculated. In the pathology, blood biochemistry, and inflammatory response tests, 24 8-week-old female BALB/c mice were placed in SPF animal room to adapt for 1 week. They were then divided into 4 groups. The sample administration method was consistent with that of the acute toxicity. Three mice were taken at 1 week and 2 weeks, respectively. The blood was collected to measure the main blood biochemical indicators, TNF-α, and IL-6 concentrations. After being euthanized, the mice were randomly selected from each group to collect heart, liver, spleen, lung, and kidney. The HE staining was performed to observe and compare pathological changes.

All animal experiments were conducted with full respect to the animal welfare and in accordance with the experimental methods approved by the Ethics Committee of Xi’an Medical University (approval document number: XYLS-2022188).

### 2.7 Construction of mouse colon cancer tumor model

4-week-old BALB/c-nu/nu mice were raised in a SPF environment for 5 days to adapt to the environment. Afterwards, 150 μL CT-26-luc or SW48 cell suspension (1 × 10^6^ cells/mL) was injected into one crotch of the mouse. After the tumors grew to a suitable size, they were used for subsequent experiments.

Six-week-old BALB/c mice were used to construct an orthotopic tumor model of colon cancer. CT-26-luc tumor-bearing mice with tumor sizes of approximately 8 × 8 mm were selected. The subcutaneous tumors were removed, and the dense tissue in the tumors was taken out and divided into small blocks of 1 × 1 mm, which were then soaked in serum-free culture medium for later use. BALB/c mice were placed in the SPF environment to adapt for 1 week. The abdominal hair was removed. The mice were given anesthesia with isoflurane gas. They were fixed on the operating table and an incision was made 1–1.5 cm above the genital tract with a scalpel. A transverse incision of 2 cm in length was made, then the peritoneum was cut open, a retractor was used to expand the incision, and the colon was exposed. The fresh tumor tissue obtained from the subcutaneous tumor was sutured to the colon wall with No. 8 absorbable sutures at approximately 1.5–2 cm from the lower end of the cecum, then the intestines were carefully placed back into the abdomen, flushed the abdominal cavity with saline containing 1% ampicillin, sutured the peritoneum and outer skin, respectively, sterilized and suture the wound, and subcutaneously injected lidocaine into the side of the wound site for analgesia. The mice were placed in clean cages for observation and were given high-nutrient feed. Their wounds were disinfected daily. Tumor formation through bioluminescence was observed using an *In Vivo* Imaging System after the wound had recovered.

### 2.8 *In vivo* distribution and ultrasound triggered release test of SCP NPs

The SW48 single-crotch tumor-bearing mice were split into two groups. IR-780 is a near-infrared fluorescent (NIF) dye with excellent imaging capabilities, which can be directly used for tracking SCP NPs *in vivo*. SCP NPs and the equivalent concentration of IR-780 were injected intravenously, respectively. The fluorescence signal distribution in mice was observed through the *In Vivo* Imaging System, and quantitative analysis was performed. The observation channels were ex: 780 nm and em: 820 nm. After the fluorescence signal *in vivo* weakened, the mice were euthanized, and the main organs and tumor tissues were collected. The fluorescence distribution and signal intensity of organs and tumor were observed under the same parameters, and the fluorescence signal was quantitatively analyzed. The CT-26-luc mouse orthotopic colon cancer model was taken and tested using the same method as mentioned above. Before testing, bioluminescence imaging is performed first to determine the tumor location.

The SW48 double-crotch tumor-bearing mice were selected and divided into two groups. SCP NPs samples and the equivalent concentration of IR-780 were injected intravenously. The left tumor area of each mouse was imaged using a small animal ultrasound system (SiliconWave, KOLO, China). The parameters were 1 MHz and 0.5 W/cm^2^. The fluorescence signal distribution was observed through *In Vivo* Imaging System at different time points. The mice were euthanized and the tumor tissues were collected. The fluorescence distribution and signal intensity were observed under the same conditions, and the fluorescence signals were quantitatively analyzed.

The SW48 single-crotch tumor-bearing mice were also divided into two groups. They were treated with ultrasound as described above. After 12 h, the mice were euthanized. The tumor tissues were embedded and sliced for immunofluorescence detection. HIF-1α expression level in tumor tissue was tested. ROS levels in the tissue were also detected using the DHE kit.

### 2.9 *In vivo* anti-tumor effect of SCP NPs

Thirty SW48 single-crotch tumor-bearing mice were selected and divided into 5 groups, namely, Saline group, MTX group, MTX + Ultrasonic group, MTX + IR-780+Ultrasonic + Laser group, and SCP NPs + Ultrasonic + Laser group. The intravenous injection dose was 1/3 of the highest tolerated concentration in the acute toxicity experiment of SCP NPs. The intervention time point was determined based on the *in vivo* imaging experiment. The ultrasound parameters were 1 MHz, 0.5 W/cm^2^, and 20 min. The laser parameters were 808 nm, 1 W/cm^2^, and 3 min. After the treatment, the tumors were observed twice a week for three consecutive weeks. The tumor volume was measured and the tumor volume change curve was drawn. The weight changes of the mice in each group were recorded at the same time. After 3 weeks, the mice that still had tumors were euthanized, and the tumor tissues were collected and observed. The weight of the tumor tissues was recorded. The remaining mice without clear tumors were observed for 14 days, and the recurrence was recorded. The mice were euthanized, the tumor tissues were collected and photographed, and the tumor weights were recorded.

### 2.10 Statistics and analysis

All experiments were repeated at least three times independently. All data were expressed as mean ± standard error (SD). The two-ways ANOVA test was used to determine whether the difference was statistically significant. *p < 0.05, **p < 0.01, and ***p < 0.001 indicate differences.

## 3 Results

### 3.1 Characteristics and performance of SCP NPs

**FIGURE 1:**
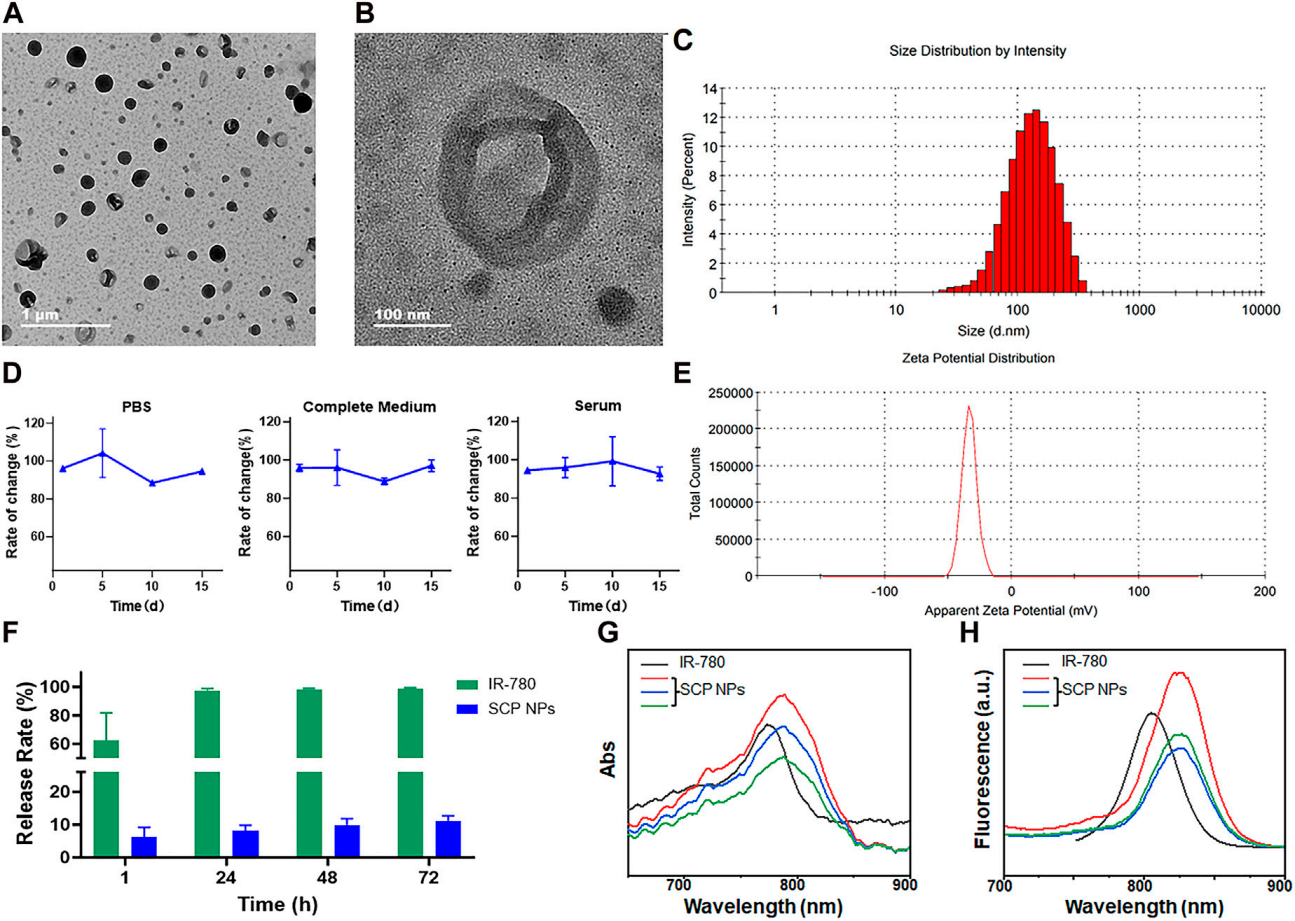
The characterization results of SCP NPs. **(A)** Full-field TEM image of SCP NPs; **(B)** Detailed TEM image of SCP NPs; **(C)** Size distribution of SCP NPs; **(D)** Size change curve of SCP NPs in PBS, complete culture medium, and serum (n = 3); **(E)** Zeta potential test results of SCP NPs; **(F)** The IR-780 release test results of SCN NPs (n = 3); **(G)** The comparison between the absorption spectrum of SCP NPs and IR-780; **(H)** The comparison between the fluorescence spectrum of SCP NPs and IR-780.

The TEM results are shown in Figure 1A. It can be seen from the figure that SCP NPs were spherical, had an obvious hollow structure, and had good monodispersity. Figure 1B is a detailed picture of SCP NPs. It can be seen that in the vacuum environment of TEM, only an empty shell was left in the nanoparticles, indicating that the outer PDA shell was completely retained. From Figure 1C, it can be seen that the hydrodynamic particle size distribution of SCP NPs was 99.7 ± 13.4 nm, and the particle size distribution was relatively wide. The main reason was that the PHF in the core of the nanoparticles was liquid, and it was difficult to achieve a highly uniform micelle size. Overall, the particle size was within 300 nm. The results of the colloidal stability test (Figure 1D) show that within 2 weeks, the particle size of SCP NPs did not change by more than 10% in PBS, complete culture medium, and serum. The zeta potential results are in Figure 1E, with an average value of −31.3 ± 3.7 mV. The above results indicated that SCP NPs basically meet the requirements for utilization *in vivo* (Shahriari et al., 2021). The encapsulation rates of IR-780 and MTX in SCP NPs were 89.12% ± 6.6% and 91.47% ± 4.8%, respectively, and the drug loading capacities were 8.71% ± 2.2% and 11.82% ± 3.7%, respectively. Within 72 h, less than 10% of IR- 780 released (Figure 1F), showing good encapsulation performance. Since SCP NPs had PT and SDT functionalities, in order to further determine whether the wrapping process would affect the optical properties of IR-780, the absorption and emission spectra were measured. The results are shown in Figures 1G, H. It can be seen that both of their absorption and the emission peaks were slightly red-shifted compared to those of free IR-780. This phenomenon often occurs in nano-encapsulated fluorescent dyes. This slight red-shift would not interfere with subsequent treatment and imaging, and even had certain benefits, such as the high absorption efficiency of the 808 nm laser (Song et al., 2019; Alves et al., 2020).

**FIGURE 2:**
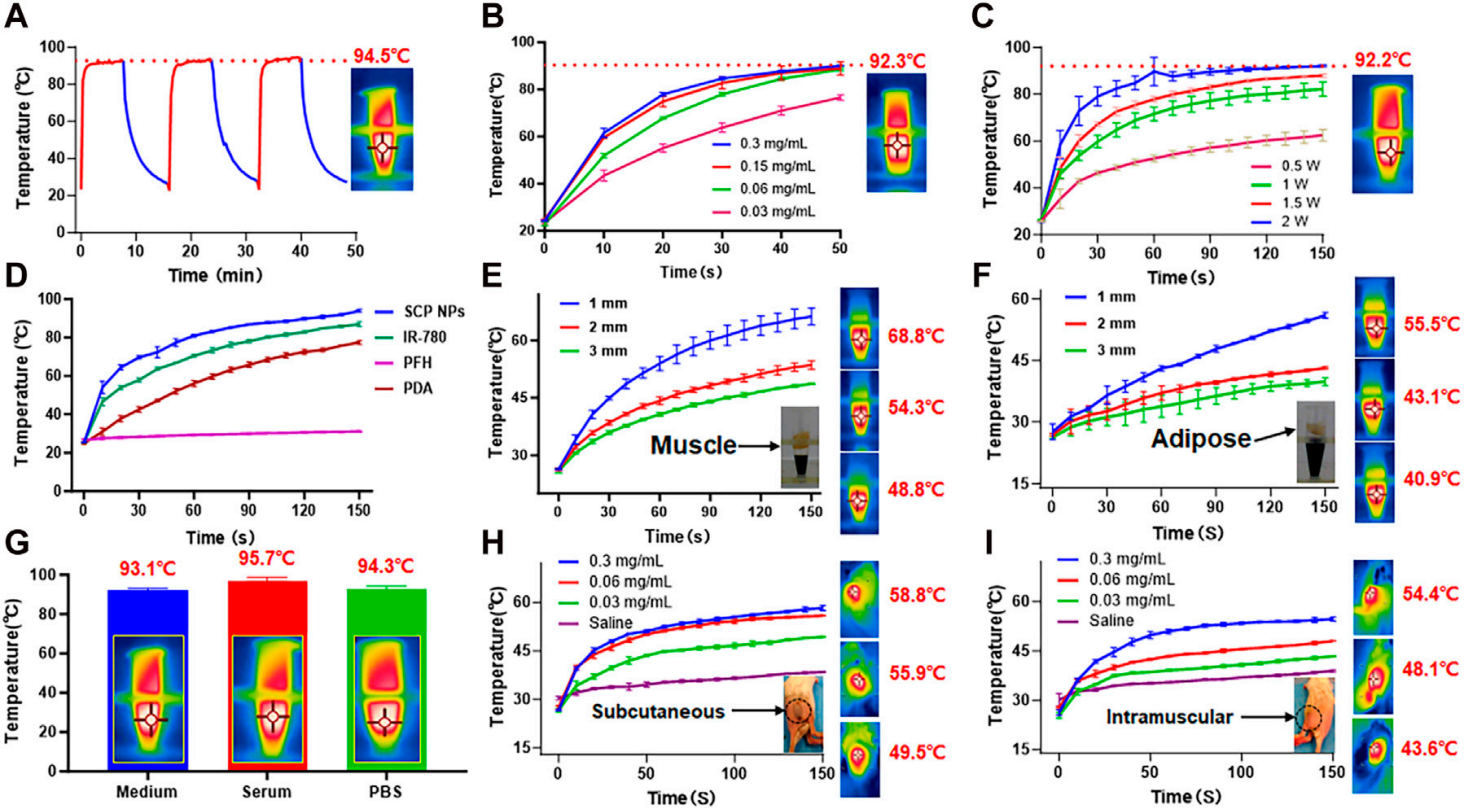
Photothermal conversion test results of SCP NPs. **(A)** Thermal stability and maximum temperature rise test results of SCP NPs, 808 nm, 2 W/cm^2^; **(B)** The temperature rise curves of SCP NPs with different concentrations under the same power laser irradiation; **(C)** The temperature rise curves of SCP NPs under different power laser irradiation; **(D)** The photothermal heating curve of SCP NPs and its main components; **(E, F)** The photothermal heating curve of SCP NPs under the blocking of muscle and fat tissue with different thicknesses, 808 nm, 1 W/cm^2^; **(G)** The maximum temperature rising test of SCP NPs in PBS, Serum, and Medium, respectively; **(H,I)** The photothermal heating curves of SCP NPs in the subcutaneous and intramuscular area of mice, 808 nm, 1 W/cm^2^. All tests were n = 3.

Subsequently, the photothermal conversion performance of SCP NPs was comprehensively tested. The first one was the photothermal stability test. From the results, it can be found that under 2 W/cm^2^ 808 nm laser irradiation, the temperature reached 94.5°C, and good heating stability was maintained for three consecutive irradiations (Figure 2A). SCP NPs also maintained stable photothermal conversion performance under different dispersion solvents and heated to very high temperatures (Figure 2G). SCP NPs also exhibited a clear dose-response relationship in terms of concentration or power, as shown in Figures 2B, C. It can be seen from the single-component temperature rise test results (Figure 2D) that in addition to the main photothermal performance coming from IR-780, PDA also played a certain role in heating. SCP NPs were designed for photothermal therapy *in vivo*, so a muscle and adipose tissues block heating experiment was conducted. From Figures 2E, F, it can be seen that the SCP NPs could effectively generate heat regardless of whether the tissue was used to block it. It is worth noting that the impact of muscle to heating was smaller than that of adipose tissue. Further photothermal conversion tests were conducted subcutaneously and intramuscularly in mice, and a very significant heating effect was observed (Figures 2H, I).

**FIGURE 3:**
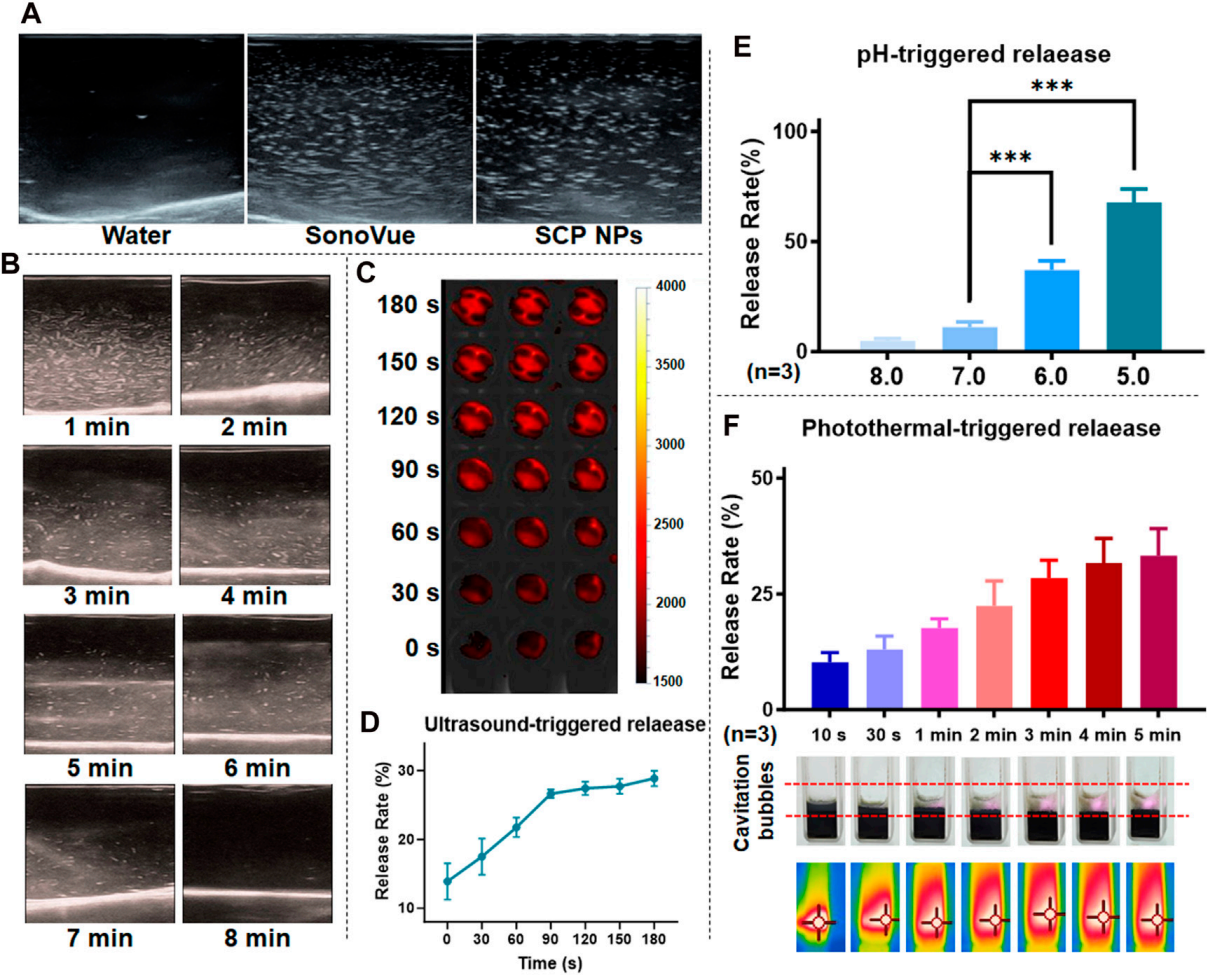
Trigger release test results of SCP NPs. **(A)** Ultrasound cavitation results of SCP NPs and ultrasound contrast agent, SonoVue in pure water; **(B)** SCP NPs ultrasound cavitation signal attenuation test results; **(C, D)** SCP NPs ultrasound triggered release results and release curves (n = 3); **(E)** SCP NPs release results at different pH (n = 3); **(F)** SCP NPs cavitation and release results under laser irradiation (n = 3). In terms of statistically significant differences, p < 0.001 is marked as ***.

We design SCP NPs to achieve controlled release under conditional triggering. First, in terms of ultrasound, SCP NPs have a cavitation effect. Compared to the clinical ultrasonic cavitation agent, SonoVue, the cavitation signals of SCP NPs appear coarser but slightly fewer in number. The results are shown in Figure 3A. The cavitation maintenance effect was then tested, and the results showed that the ultrasonic signal of SCP NPs gradually weakened over time, and the signal disappeared around 8 min, indicating that they have a faster triggered release effect (Figure 3B). IR-780 in SCP NPs was wrapped in PDA, causing its fluorescence to be quenched (Qiang et al., 2014). Based on this, the study tested its triggered controlled release through the fluorescence intensity after release. The results of the ultrasonic triggered release test and quantitative analysis are shown in Figures 3C, D. It can be found that as the ultrasonic time went on, the fluorescence signal of the sample became stronger, and the upward trend of the quantification curve could also be seen. Figure 3E is the release effect of SCP NPs in different pH environments. It can be seen that as the pH decreased, the release rate of IR-780 gradually increased, indicating that SCP NPs can effectively release the payloads in the acidic environment of tumor tissues and cells. Finally, the photothermal triggered release of SCP NPs was tested (Figure 3F). It can be seen that with the laser irradiation, the sample temperature continued to increase, obvious foaming phenomenon was observed, and the release rate also gradually increased. These results indicate that SCP NPs have good responsive release ability.

### 3.2 Cytotoxicity and combined intervention effects of SCP NPs *in vitro*

**FIGURE 4:**
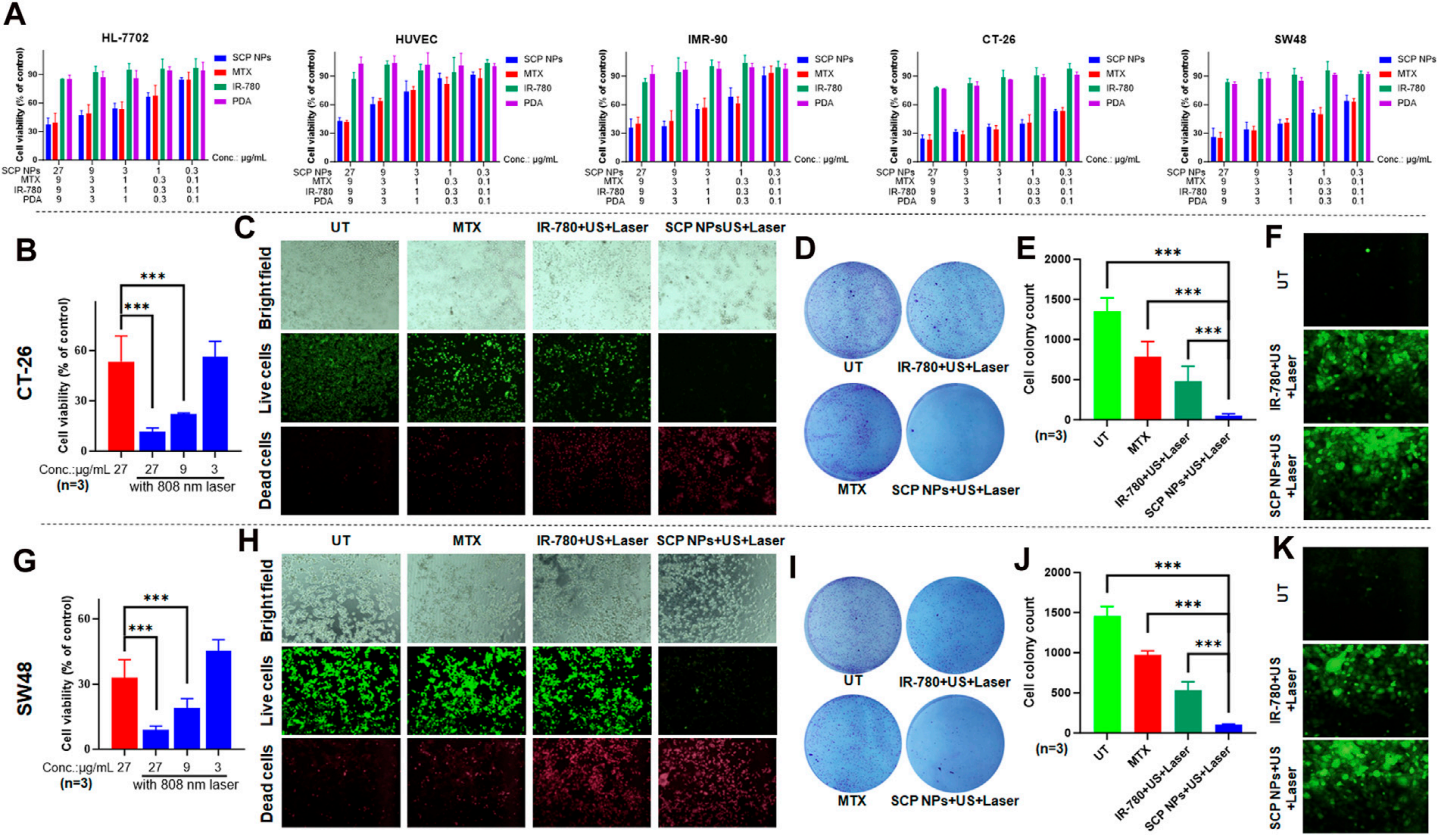
Synergistic therapeutic effect of SCP NPs at the cellular level. **(A)** Cytotoxicity test results of SCP NPs, the cell lines were HL-7702, HUVEC, IMR-90, CT-26, and SW48; **(B)** Cell survival rate of CT-26 cell line under photothermal treatments (n = 3); **(C)** The fluorescence microscope images of CT-26 cells with live and dead cell staining after the treatment of SCP NPs combined intervention; **(D, E)** The colony formation results of CT-26 cells after the treatment of SCP NPs combined with different interventions (n = 3); **(F)** The fluorescence microscope images of CT-26 cells’ intracellular ROS production with different interventions; **(G)** The cell survival rate of SW48 cells after the SCP NPs and photothermal treatments (n = 3); **(H)** The fluorescence microscope images of SW48 cell with live and dead cell staining after the treatment of SCP NPs combined with intervention; **(I, J)** The colony formation results of SW48 cells after the treatment of SCP NPs combined with different interventions (n = 3); **(K)** The fluorescence microscope images of SW48 cells’ intracellular ROS production with different interventions. In terms of statistically significant differences, p < 0.001 is marked as ***.

We used 3 normal cell lines and 2 colon cancer cell lines to detect the *in vitro* cytotoxicity of SCP NPs. As shown in Figure 4A, PDA and IR780 did not have obvious cytotoxicity, while the toxicity of MTX continued to increase as the concentration increased. It is worth noting that SCP NPs showed a similar cytotoxic effect to the MTX group, indicating that it can effectively exert treatment effect of MTX.

The synergistic effect of SCP NPs at the cellular level was then evaluated through CCK-8, live and dead cell staining, colony formation, and ROS generation tests. The mouse and human colon cancer cell lines CT-26 and SW48 were selected. It can be seen from Figures 4B, G that SCP NPs plus laser irradiation had obvious differences. At the same concentration, the cell survival rate decreased by 5 times and 3 times, respectively. Even if the nanoparticle concentration was reduced to 1/3, there was still a stronger treatment effect. Further refinement was made in the cell treatment groups: chemotherapy alone, IR-780+PT + SDT, and SCP NPs + PT + SDT. Live and dead cell staining and colony formation tests were conducted to evaluate the effect. The results are shown in Figures 4C–E (CT-26), Figures 4H–J (SW48). It can be seen that chemotherapy alone had a treatment effect, but the effect was not significant due to the low dose. PT + SDT had a better effect, but there were still a large number of cells survival. The cell death rate increased greatly under SCP NPs combined with PT and ultrasound, with only a few cells survived, showing a very strong synergistic effect. In order to further prove the synergistic effect of SCP NPs, the indicator ROS was selected since both PT and SDT are related to intracellular ROS levels. Figures 4F, K are fluorescence micrographs of ROS produced by 2 cell lines under different intervention methods. It can be clearly seen that SCP NPs can effectively reflect the PT and SDT effects. Compared with the control group, they produced a large amount of ROS in different cell lines, and the fluorescence intensity of SCP NPs was slightly higher than that of the IR-780 group. These results all proved that SCP NPs can achieve the synergistic intervention effect of PT, SDT, and chemotherapy.

### 3.3 Cellular internalization and affinity test results of SCP NPs

**FIGURE 5:**
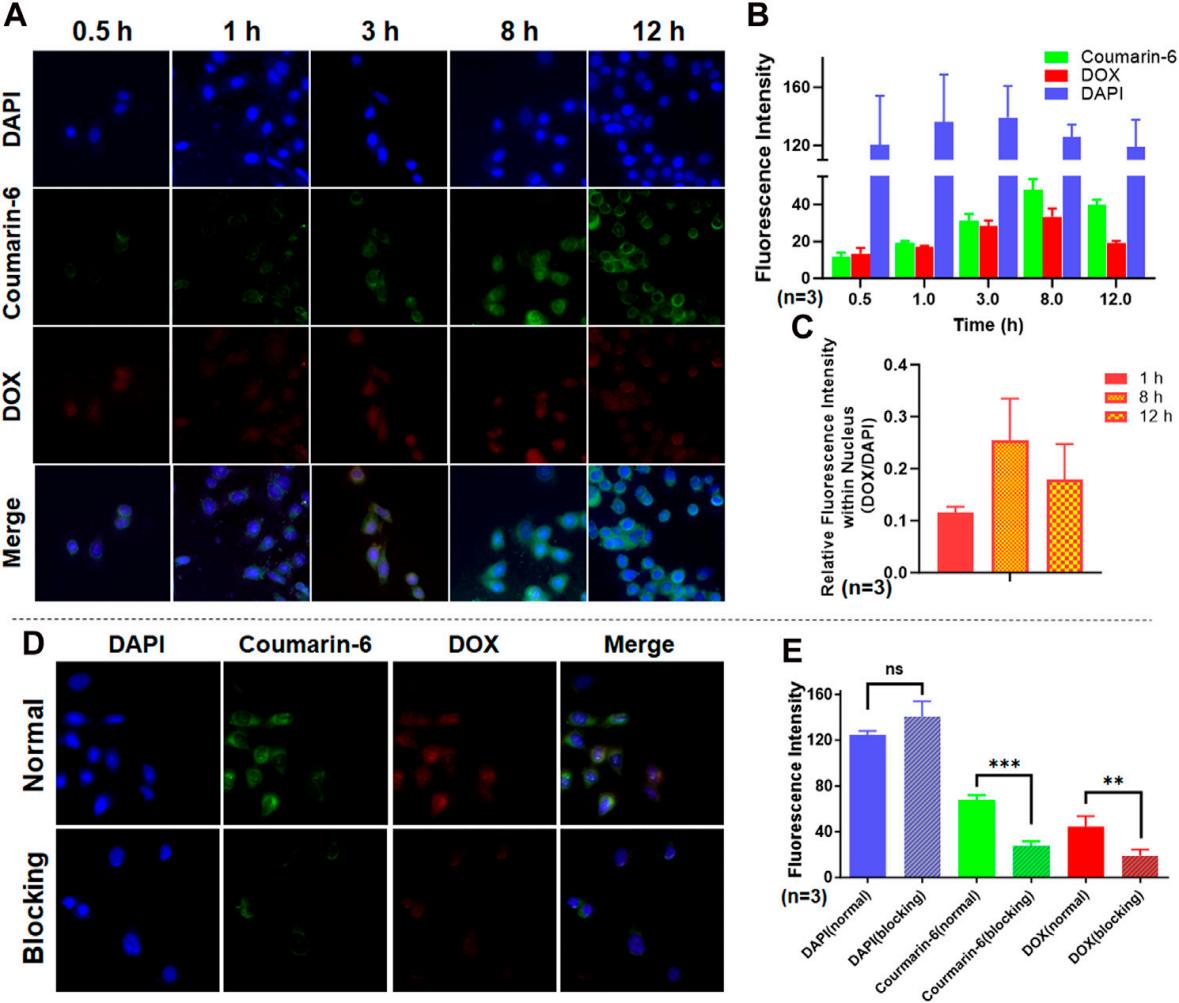
Cell internalization and affinity test results of SCP NPs. **(A)** The fluorescence signal accumulation after SCP NPs were endocytosed by SW48 cells; **(B)** The quantitative results of three fluorescence signals in cells (n = 3); **(C)** The comparison of relative fluorescence intensity of DOX and DAPI in the nucleus during the experiment (n = 3); **(D)** The fluorescence microscope images of SCP NPs internalization in SW48 cells after endocytosis was inhibited by NaN_3_; **(E)** The comparison of three fluorescence signal intensities (n = 3). In terms of statistically significant differences, non-significant differences are marked as ns, p < 0.01 is marked as **, and p < 0.001 is marked as ***.

SCP NPs were first labeled with two fluorescent dyes and the cell internalization tests were performed. The results are shown in Figure 5A. It can be seen from the fluorescence micrographs that the blue fluorescence signal of DAPI labeling the across groups was basically the same. In contrast, the green and red fluorescence intensity within the cell becomes increasingly stronger over time. After further quantification (Figure 5B), the overall intracellular fluorescence had remained at a high level from 8 to 12 h, and the signal of DOX had decreased at 12 h. This may be related to the properties of the two fluorescent molecules. Coumarin-6 is a hydrophobic molecule that is mainly bound to various membrane structures in cells. DOX is a hydrophilic molecule that is easily excreted by cells. Hence, we further analyzed the relative fluorescence signal (DOX/DAPI) on the cell nucleus and found that the DOX signal on the cell nucleus was much stronger at 8 h than at 12 h (Figure 5C), indicating that DOX was gradually released from SCP NPs to the cytoplasm and entered the nucleus. To prove that fluorescent molecules were indeed delivered into cells by SCP NPs through active transport, we pretreat the cells with an active transport inhibitor, NaN_3_, and then added fluorescent SCP NPs for co-incubation. The results showed that after adding the inhibitor, the cells The fluorescence signal inside was significantly reduced, and the microscopic imaging results and fluorescence intensity quantitative analysis results are shown in Figures 5D, E respectively.

### 3.4 SCP NPs biocompatibility test results

**FIGURE 6:**
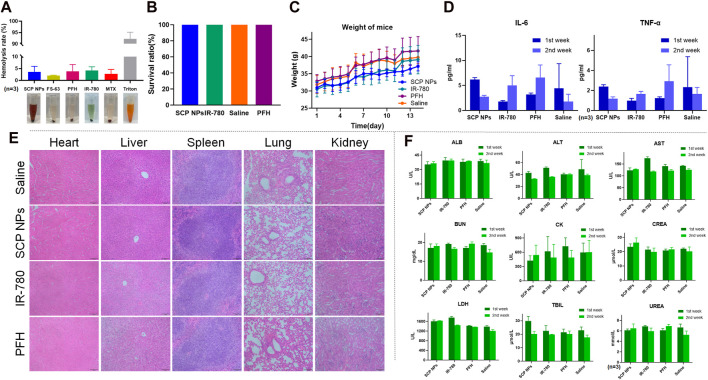
Biocompatibility test results of SCP NPs. **(A)** The hemolytic test of SCP NPs and its main components (n = 3); **(B)** The survival rate of mice in each group in the acute toxicity test; **(C)** The average body weight change curve of mice in each group in the acute toxicity test; **(D)** The concentrations of inflammatory factors TNF-α and IL-6 in mice blood in each group during the first and second weeks of acute toxicity test (n = 3); **(E)** The HE staining results of the main organ tissues of mice in each group during the first week of acute toxicity test; **(F)** The main blood biochemical indicators of mice in each group in the first and second weeks of acute toxicity test (n = 3).

Biocompatibility is a core indicator of SCP NPs. First, the impact of SCP NPs on red blood cells was evaluated through hemolysis experiments (Figure 6A). It can be seen from the hemolysis rate that the positive control Triton X-100 caused severe hemolysis, with more than 90% of red blood cells damaged. The hemolysis rates in other treatment groups were relatively low, and no obvious hemolysis occurred in SCP NPs or their components treatment groups, indicating a good injection safety of SCP NPs.

The acute toxicity of SCP NPs was then tested using BALB/c mice. The injection dose was 50 mg/kg. Within 14 days, the mice remained alive without obvious toxic reactions, and their body weight continued to grow steadily. The results are shown in Figures 6B, C. We then further evaluated whether SCP NPs could cause the immune, physiological, pathological reactions in major organs of mice. In terms of inflammation, the levels of TNF-α and IL-6, the two most representative immune factors in the serum of mice, were detected for two consecutive weeks. The results are shown in Figure 6D. The levels of TNF-α and IL-6 in SCP NPs group dropped in the second week after injection. In the control group, although the levels of both inflammatory factors increased in the second week, the concentrations of both factors were at relatively low levels. The results showed that SCP NPs and their main components did not cause a clear immune response in the body. From histological observation of the main organs, we found that there were no obvious pathological changes in the main organs of mice in all groups after 1 week (Figure 6E). The only difference was that there was a slight increase of lymphocytes in the livers of mice in the SCP NPs group, but other pathological features such as binucleates and megakaryocytes were rare. Another histological observation was conducted 2 weeks later. The results are shown in Supplementary Figure S1. No obvious pathological changes were found. The blood biochemical test results are shown in Figure 6F. It can be seen that the test results of most indicators in the first and second weeks did not show obvious differences. However, the CK values of all mice were high, indicating that there might be myocardial damage. Other indicators were all in the normal range. Taken together, SCP NPs demonstrated very good biocompatibility and can be safely used for subsequent *in vivo* imaging and therapeutic experiments.

### 3.5 *In vivo* distribution and NIF imaging results of SCP NPs

**FIGURE 7:**
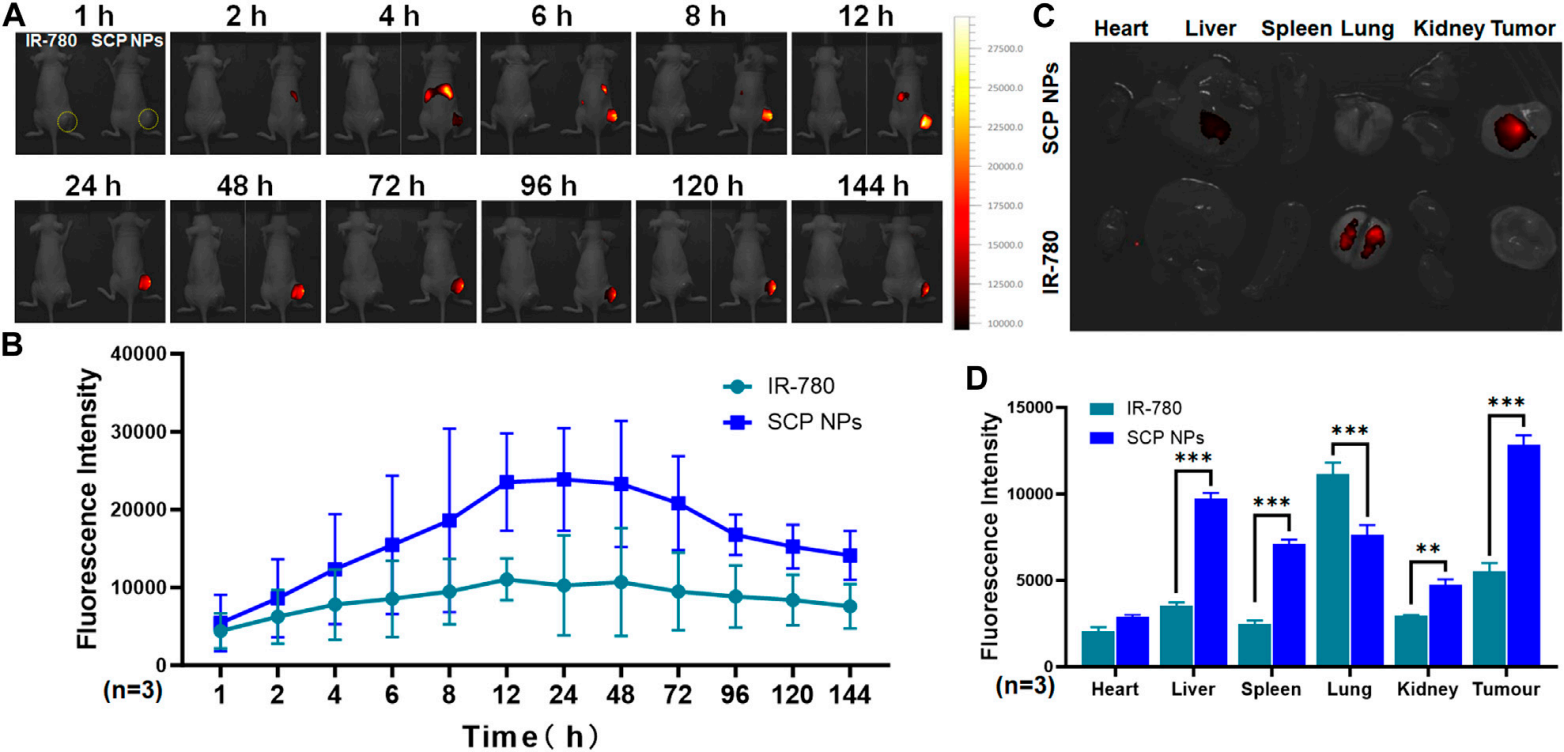
*In vivo* distribution and targeted delivery performance of SCP NPs in SW48 mouse subcutaneous tumor model. **(A)** The fluorescence signal distribution of SCP NPs in mice. At each time point, the left and right mice were injected with IR-780 and SCP NPs, respectively. The yellow dotted line marks the tumor area; **(B)** The quantitative analysis of fluorescence signals in the tumor area (n = 3); **(C)** The residual fluorescence signals distribution of major organs and tumor of mice; **(D)** The quantitative analysis of fluorescence signal intensity of major organs and tumor of mice (n = 3).

First, the modeled SW48 single-tumor mice were randomly divided into two groups for SCP NPs and IR-780 injections. SCP NPs and IR-780 were injected intravenously. The concentrations of IR-780 in both groups were the same. The continuous dynamic observation results are shown in Figure 7A. The mouse on the left and right were injected with IR-780 and SCP NPs, respectively. The tumor is outlined by yellow dotted lines. It can be seen that under the same observation parameters, the signal of the IR-780 group is significantly weaker than that of the SCP NPs group. The fluorescence signal of the mice in the SCP NPs group was mainly concentrated in the spleen and liver areas in the first 4 h, and in the tumor area after 4 h. The fluorescence signal gradually accumulated, then rose rapidly, and reached the highest peak at 12 h. The high fluorescence signal continued to be maintained until 48 h, and then the fluorescence signal at the tumor site was maintained for more than 144 h, indicating that SCP NPs could not only achieve long-term circulation in the body, but also be independently targeted and delivered to the tumor area. The fluorescence quantitative analysis of the tumor area is shown in Figure 7B. It can be seen that the high signal period was from 12 h to 48 h. Except for the time point right after injection, the signals at all the other time points were significantly stronger than those of the IR-780 group. The mice were then euthanized and the main organs and tumor tissues were collected and the fluorescence intensity was calculated. The results are shown in Figures 7C, D. It can be seen that except for the lungs, the signals of various organs in the SCP NPs group were higher than those in the IR-780 group. This may be because IR-780 is a hydrophobic molecule that clumped after entering the body to form large particles and accumulated in the lungs. The fluorescence signal in the tumor and liver was still high after 1 week. This scenario and the high signal phenomenon in the abdominal area during the previous *in vivo* imaging process illustrated that SCP NPs can achieve long circulation and slow release in the body.

**FIGURE 8:**
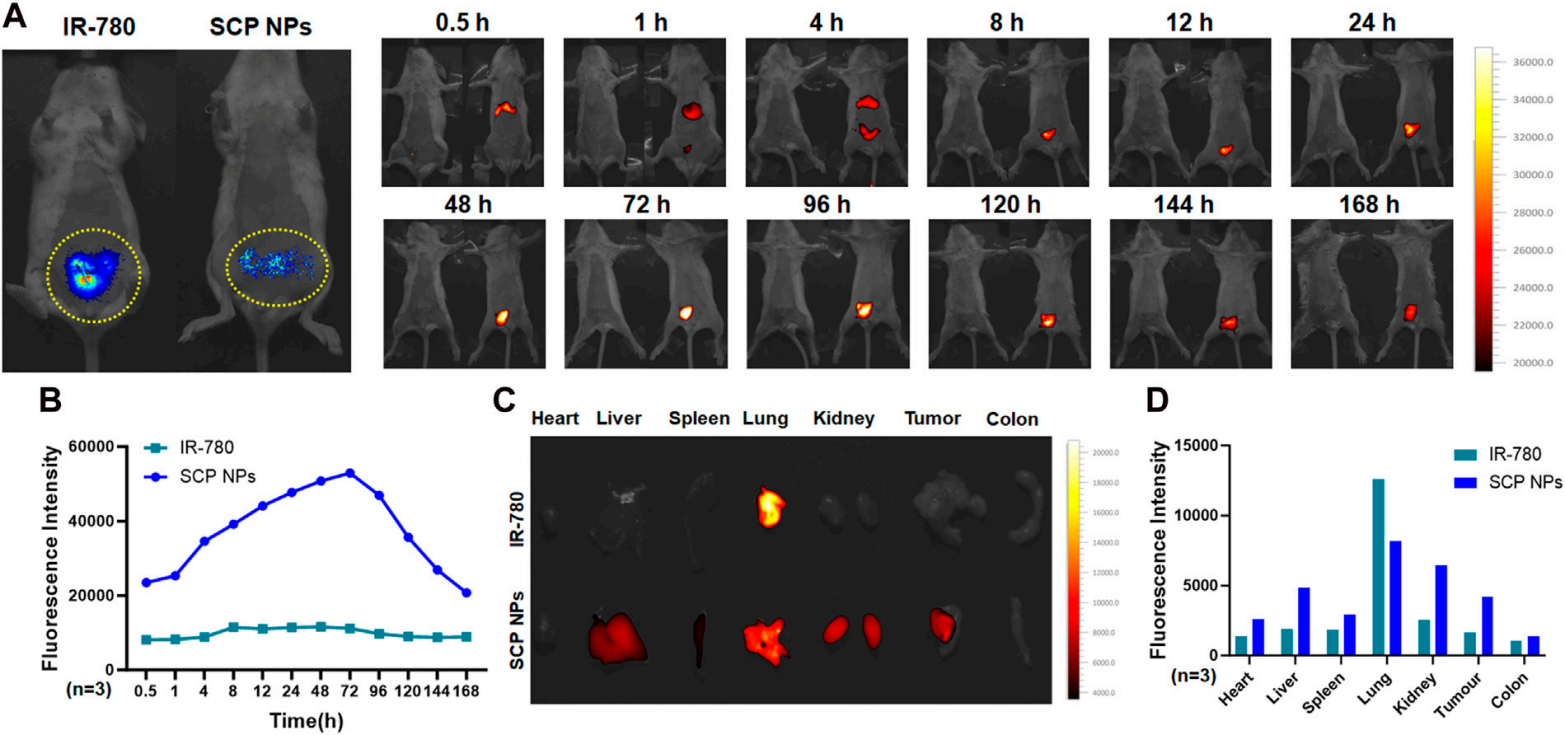
*In vivo* distribution and targeted delivery performance of SCP NPs in the CT-26-luc mouse colon cancer orthotopic tumor model. **(A)** The left photo shows the bioluminescent imaging of tumors. The yellow dotted line on the lower abdomen is the modeling area. Obvious signals can be seen in the circle. The right photos show the fluorescence of SCP NPs in mice; **(B)** The quantitative analysis results of fluorescence signal in mouse tumor area (n = 3); **(C)** The distribution of residual fluorescence signals in major organs and tumor of mice; **(D)** The quantitative analysis of fluorescence signal intensity in major organs and tumor of mice (n = 3).

The NIF effect of SCP NPs was evaluated on the colon cancer CT-26-luc orthotopic model. The results are shown in Figures 8A,B. The far-left side is the bioluminescence imaging result of the colon cancer tumor site. It can be seen that there is a clear signal in the lower abdomen, indicating the colon cancer orthotopic model construction was successful. Subsequently, the SCP NPs and the equivalent concentration of IR-780 were injected using the same method described previously, and the fluorescent signal appeared in the upper abdomen of the mouse half an hour after the injection and lasted until 4 h, which was consistent with the results in the subcutaneous tumor model. After 4 h, a clear fluorescence signal appeared in the lower abdomen of the mice in the SCP NPs group. As time went by, the signal intensity continued to increase, reaching a high value at 24 h, and continued to increase until 72 h, and then slowly decreased. The fluorescence signal of tumor was maintained until 168 h. This result also proved the excellent *in vivo* stability of SCP NPs. SCP NPs can greatly extend the *in vivo* circulation time of IR-780 and effectively achieve imaging of tumor sites, achieving a dynamic observation functionality in the subsequent experiments. After the mice were euthanized, the residual signals in major organs and tumors were detected. The results are shown in Figures 8C, D. The mice injected with IR-780 still had more signal in the lungs. In contrast, the signal in SCP NPs-treated group was mainly accumulated in tumor, liver, lungs and kidneys. These results fully demonstrated that SCP NPs can prolong the circulation time of drugs, and can be effectively accumulated in colon cancer tumors, realizing good NIF imaging effects.

### 3.6 *In vivo* ultrasound-triggered release, imaging and SDT effects of SCP NPs

**FIGURE 9:**
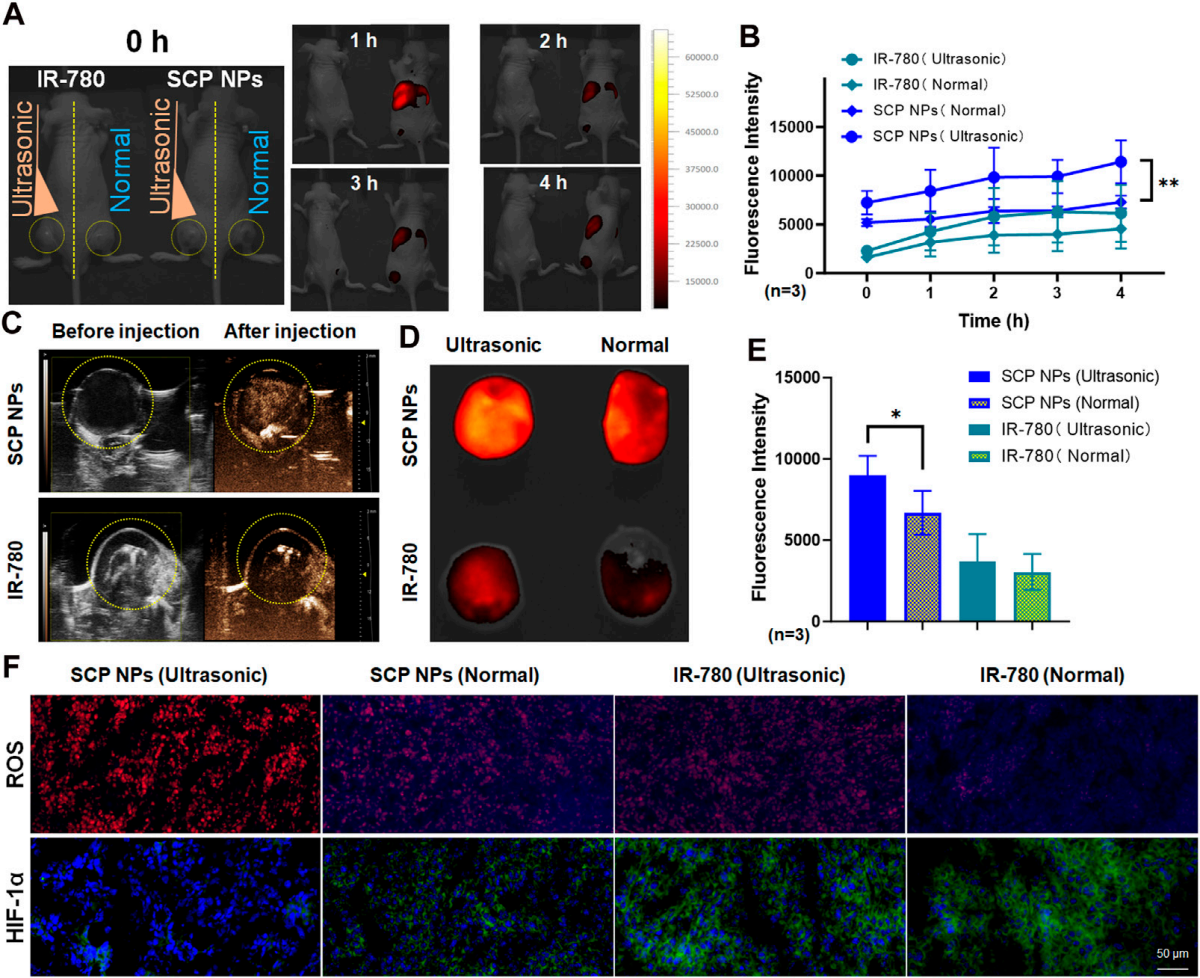
*In vivo* ultrasound triggering and SDT test results of SCP NPs. **(A)** The fluorescence signal distribution in mice after SCP NPs were triggered by ultrasound. The left and right mice were injected with IR-780 and SCP NPs, respectively. The left tumors were treated with ultrasound. The right tumors are non-triggered. The yellow dotted line marks the tumor area; **(B)** The quantitative analysis results of fluorescence signals in the tumor areas (n = 3); **(C)** The ultrasonic imaging results of the SCP NPs and IR-780 injected mice with ultrasound triggering one side of the tumors, the yellow dotted line marks the tumor area; **(D)** The distribution of retained fluorescence signal in the tumors; **(E)** The quantitative analysis of fluorescence signal intensity in the tumors (n = 3); **(F)** The fluorescence images of ROS generation levels and immunohistochemical analysis results of HIF-1α of the tumor in different treatment groups of mice. In ROS detection, DHE exhibits weak blue fluorescence, while its reaction products with ROS emit strong red fluorescence. In HIF-1α immunohistochemical staining, cell nuclei are labeled with blue fluorescence from DAPI, whereas HIF-1α is marked with green fluorescence by antibodies. In terms of statistically significant differences, p < 0.05 is marked as * and p < 0.01 is marked as **.

Double-crotch tumor model mice were selected to evaluate the *in vivo* triggered release and imaging effects of SCP NPs. The left and right mice were injected with free IR-780 and SCP NPs, respectively, with the same concentrations of IR-780. Mice were treated with ultrasound only to the left tumor area. Figure 9A shows *in vivo* fluorescence imaging after ultrasound triggering. It can be seen that the fluorescence signal on the IR-780 treated mouse was very weak, while the fluorescence signal of SCP NPs treated mouse quickly accumulated in the liver and spleen, and the intensity was higher than that of IR-780 group. The signal was then accumulated in the tumor tissue. The signals in the left and right tumors had a significant difference and became higher with more irradiations. The tumor on the other side that did not receive ultrasound irradiation had no obvious fluorescence signal. The fluorescence intensity of the tumor areas of two mice was quantitatively analyzed, and the results (Figure 9B) further confirmed the advantages of ultrasound triggering. In addition, ultrasound imaging (Figure 9C) of the tumors on the triggered side before and after injection showed that obvious cavitation signals were generated in the tumors of the SCP NPs treated mouse. Afterwards, the mice were euthanized, and the tumors were collected for fluorescence photography and quantitative analysis of fluorescence intensity. The results are shown in Figures 9D, E. In the SCP NPs group, the tumor fluorescence signal on the ultrasound-triggered side was significantly higher than that of the non-triggered side. There is no significant difference in signal of both sides of IR-780 treated group, but the fluorescence signal on the ultrasound side is slightly higher, indicating that ultrasound treatment can help drug molecules accumulate in the tumor area. These results fully demonstrated that SCP NPs have excellent ultrasonic-triggered release properties.

Afterwards, the HIF-1α expression and ROS production level in the tumors were detected (Figure 9F). DHE itself exhibits weak blue fluorescence, until it is oxidized to ethidium by ROS, which then intercalates into DNA, making the cell nuclei appear bright red. It can be seen that, in the IR-780 group, the ROS level of the ultrasound-treated tumor was slightly higher than that of the non-treated tumor. This result is in consistent with the results at the cell level, indicating that IR-780 has a certain SDT effect. It is noteworthy that in the SCP NPs group, the ROS level of the ultrasound treated tumor was significantly higher than that in the other groups, and the blue background of the DHE substrate in the group was almost invisible, fully proving that SCP NPs’ excellent SDT Effect. There were also significant differences in the expression of HIF-1α, which was lower in the SCP NPs group and higher in IR-780 groups, indicating that the nanoparticles delivered oxygen to the tumor to relieve hypoxia. The expression of HIF-1α in the two groups treated with ultrasound was lower than that in the non-treated group, suggesting that ultrasound treatment can increase the degree of dissolved oxygen in tissues and also has a certain effect in alleviating hypoxia. These results clearly proved the combined intervention of SCP NPs.

### 3.7 *In vivo* anti-tumor results of SCP NPs

**FIGURE 10:**
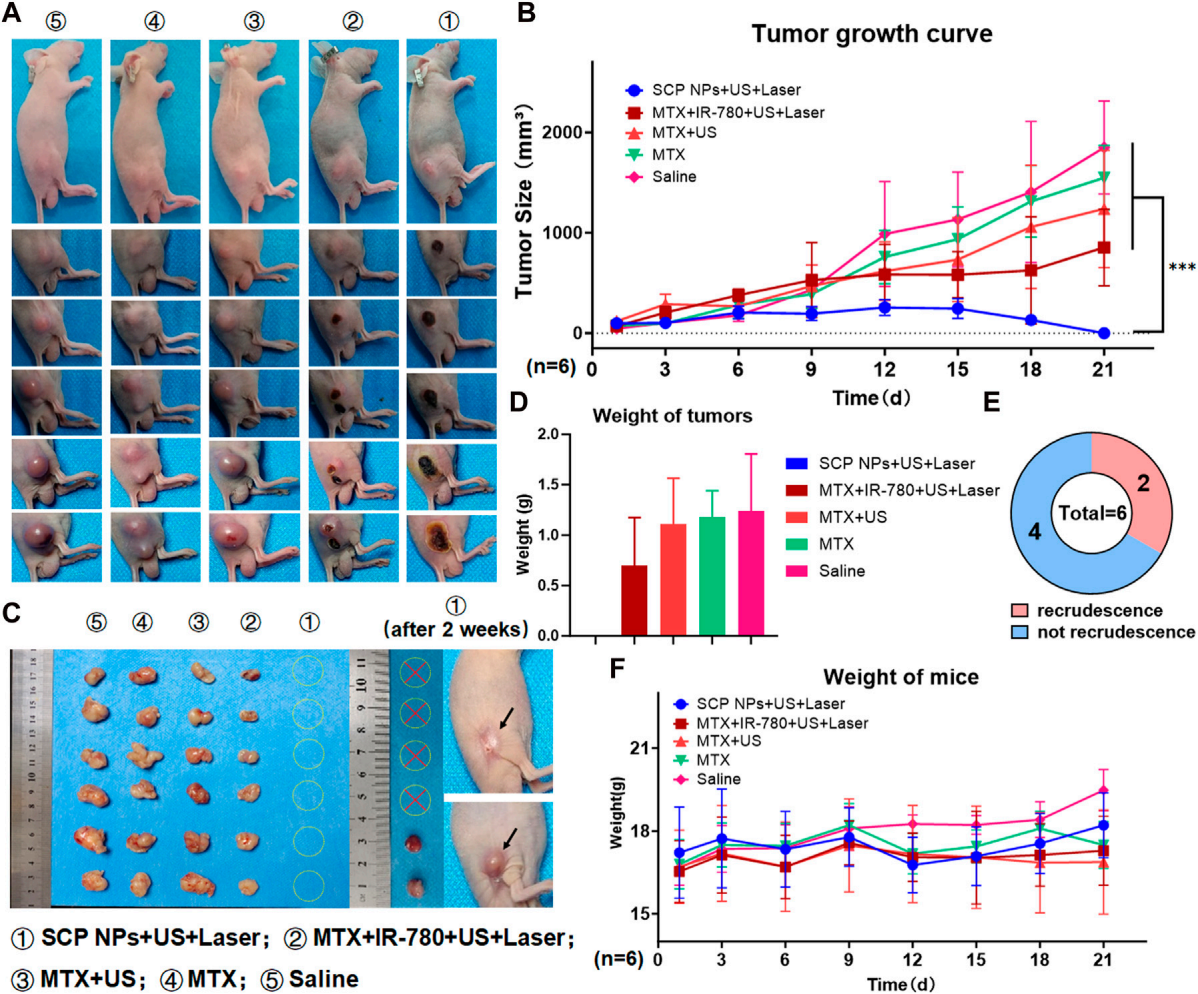
*In vivo* anti-tumor test of SCP NPs. **(A)** The photos of mice’s tumor areas in each group during the test; **(B)** The average tumor volume growth curve of mice in each group during the intervention; **(C)** The visible residual tumor in each group of mice after the intervention, and the tumor recurrence of SCP NPs combined treatment mice 2 weeks after the intervention. The arrows point to the recrudescent tumor and recovery area; **(D)** The analysis results of the visible residual tumors’ average weight in each group of mice after the intervention; **(E)** The statistics of tumor recurrence for mice in the SCP NPs combined treatment group 2 weeks after the intervention; **(F)** The average weight change curve of mice in each group during the intervention process.

To verify the *in vivo* antitumor effect of SCP NPs, we established a subcutaneous tumor model using the human colon cancer cell line SW48 in BALB/c-nu/nu mice. Specifically, a 150 μL SW48 cell suspension at a concentration of 1 × 10^6^ cells/mL was injected subcutaneously into the right flank of the mice. When the average tumor volume reached approximately 100 mm³, the mice were randomly grouped for antitumor efficacy testing. The administration method of SCP NPs is tail vein injection, the dose is approximately 8 mg/kg. Based on the *in vivo* distribution results, we chose to conduct the combination treatment within the 12–24 h period after intravenous injection. Under the same power, the maximum surface temperature of the tumor during Laser irradiation in the SCP NPs group was about 43°C. The duration was approximately 2 min. During the experiment, mice in each group were photographed, and the results are shown in Figure 10A. The tumor volumes of each group at different time points were plotted to obtain the tumor growth curve (Figure 10B). It can be seen that at first, the tumors were of the same size. As time went by, the tumors in the control group increased rapidly. Especially after 10 days, the growth accelerated. The tumors in both chemotherapy groups continued to grow. The tumors in the MTX group with ultrasound treatment were slightly smaller than those in the MTX alone group, but there was no significant difference between the two groups. The tumor growth in the MTX + IR-780 + ultrasound + laser group were obviously inhibited. It can be seen that the tumors also ruptured from the inside out. The tumor growth rate slowed down significantly after 10 days, but at a later stage, the tumors can no longer be effectively inhibited and showed a trend of accelerated growth. It is worth noting that the SCP NPs + ultrasound + laser group showed a very strong intervention effect. After the second intervention, the tumor became congestion, and then gradually began to rupture from the inside, and the rupture was consistently maintained on the surface of the entire tumor area. On the 21st day, only scabs remained on the crotches of 6 mice, and no visible tumor tissue was found. The tumor growth curve of this group also gradually dropped to almost incalculable after 2 weeks. The tumor tissue was collected and photographed. The results are shown in Figure 10C. It can be seen that the tumors in the Saline, MTX, and MTX + US groups were relatively large and irregular, while the tumors in the MTX + IR-780+US + Laser group were smaller than those of the three above-mentioned groups, and the tumor shape was relatively regular, indicating that the treatments have a certain tumor intervention effect, but cannot effectively inhibit. The results are shown in Figure 10D. The SCP NPs + US + Laser group could not be weighed because there was no obvious tumor. After that, the 6 mice in this group were continued to be raised. After 14 days, we observed that the scabs at the tumor site of 4 mice fell off and the wounds had completely healed. The mice were in good condition. The other two mice had tumor recurrence. The results are shown on the right side of Figure 10C. As shown in Figure 10E, after one continuous intervention, the cure rate of the SCP NPs group was 67%. During the entire experiment, the weight of mice in each group was also monitored. The results are shown in Figure 10F. The weight of mice in the SCP NPs group began to gradually increase after 12 days, further proving the *in vivo* safety of SCP NPs.

## 4 Discussion

The heterogeneity of colon cancer often leads to over- or under-treatment. At present, single treatment methods for colon cancer have limitations such as poor efficacy and insufficient prognosis. Orderly combined treatment is currently a more recognized development idea. The essence of combined treatment is the reasonable combination of multiple methods, mutual support, and synergistic effect. PT, including PDT and PTT, relies on light irradiation to induce tumor cell death (Han et al., 2019; You et al., 2020; Li et al., 2022b; Song et al., 2022). Moreover, studies have shown that PT and chemotherapy have a good synergistic anti-tumor effect (Jiang et al., 2017; Shao et al., 2017; Zeng et al., 2018). Nanocarriers can load different therapeutic molecules based on needs, and achieve synchronous delivery to tumor tissues and cells through targeted delivery and responsive release, effectively exerting a synergistic effect (Xie et al., 2020). The main problem of PT is that the tissue penetration is limited, and can only be used for epidermal or superficial tumors. However, gastrointestinal tumors including colon cancer can also be reached with optical fiber to achieve laser irradiation of the tumor site. Additionally, to solve the problem of insufficient penetration depth of light irradiation, SDT was developed and used in the treatment of various diseases including tumors (Chen et al., 2014). But the generation of ROS in SDT and PDT often stops with the depletion of oxygen. For this reason, some scholars have constructed an intelligent nanoplatform with O_2_ self-supply capability to reverse unfavorable hypoxic conditions, reduce the expression of HIF-1α, and promote anti-tumor effects (Zhu et al., 2022). However, this type of nanocarriers also has many disadvantages, such as complex preparation and poor metabolism of the materials used. How to rationally design a nanocarrier that is simple to prepare and meets various needs is the essence of integrating the above functions.

Zhu et al. designed a nanocarrier with PFH core and a PEI-PDA shell. The drug can be quickly released through ultrasound treatment (Zhu et al., 2019). But this carrier has not been used in research on tumor treatment. Based on this carrier, we designed a nanocarrier that can achieve cascade-triggered release of PTT-chemotherapy combined therapy, achieving the simultaneous accumulation of two molecules with different physical and chemical properties to the tumor area, realizing a synergistic treatment. However, this carrier can only be used for superficial tumors such as breast cancer due the low penetration depth of light radiation (Li et al., 2021b). Based on the previous study, we further integrated the SDT with LUS, and used the high oxygen-carrying properties of PFH to effectively alleviate the hypoxic nature in the tumor tissue and achieve an excellent therapeutic effect. The prepared SCP NPs have a particle size of approximately 100 nm, good dispersion, and a negatively charged surface. SCP NPs maintained good colloidal stability in different solutions. The *in vivo* distribution results also proved that they have excellent stability in the body, remaining aggregating in tumor for more than a week after one injection in both subcutaneous and orthotopic tumor models. On the other hand, SCP NPs also have a clear multiple-triggered release effect, achieving drug release within tumor tissues and cells. The PFH carried by the nanoparticles can stay stably inside the particle carrier and can cause cavitation effect under ultrasound. There were no obvious differences with the commercial ultrasound contrast agent, SonoVue, and SCP NPs. SCP NPs’ release rate was even faster than SonoVue. This result showed that the ultrasound can quickly rupture the nanocarriers to release the drug, and also intensify the tissue’s absorption of drug molecules, achieving simultaneous and efficient delivery (Canavese et al., 2018; Awad et al., 2021). PDA has a pH-responsive release effect and can degrade in tumor microenvironment. Therefore, SCP NPs can achieve drug release within tumor tissues and cells. SCP NPs also have a heat-triggered release effect, which can gradually release the drug during the heating process caused by laser radiation. These results together contribute to SCP NPs’ good drug delivery performance, enabling more therapeutic molecules to accumulate in the tumor area, enhancing the therapeutic effect, reducing the frequency and dosage of administration, and reducing the side effects and drug resistance. The biocompatibility test also proved that the toxic side effects of SCP NPs are minimal. From the cell test, it can be seen that the nanoparticles showed an inhibition due to chemotherapy drugs, but did not show excessively strong cytotoxicity. The safety of the nanoparticles was further proved in the *in vivo* toxicity test. After high-dose injection, the mice did not have pathological reactions, let alone death. There were no pathological changes in the main organs. The inflammation levels and blood biochemical results showed no abnormalities.

The synergistic effect of the combined treatment of PTT, chemotherapy, and SDT is the core property of SCP NPs. We tested the synergistic therapeutic effect of SCP NPs from many aspects, and the results were gratifying, effectively proving the excellent therapeutic effect of the nanoparticles. SCP NPs have good encapsulation performance. Due to the unique encapsulation method of PDA, SCP NPs will not change the molecular structure of the encapsulated material and retain the activity to the maximum extent. During the test, it was found that the spectral characteristics of IR-780 did not change significantly. In terms of the photothermal conversion, the maximum temperature rise of SCP NPs in aqueous solution exceeded 92°C, and there was no obvious change in photothermal conversion in different solutions. Muscle and fat blocking will reduce the temperature rise, but it can still meet the needs of hyperthermia, and ensure that the temperature rises to an effective temperature in mice. This heating effect is also excellent compared with other IR-780 related nanocarriers (Chen et al., 2016; Hu et al., 2021). At the cellular level, human and mouse colon cancer cell lines were tested respectively. As a chemotherapy drug, MTX has complementary mechanisms of action with ROS and hyperthermia. Firstly, both ROS and hyperthermia can cause DNA damage, and MTX inhibits DNA synthesis, causing cells to be unable to effectively repair DNA. Secondly, both ROS and hyperthermia can cause cell apoptosis. MTX is also an apoptosis inducer, and the combination of MTX, hyperthermia, and ROS can aggravate cell apoptosis. The experimental results fully demonstrate that SCP NPs effectively retained the cell inhibitory ability of MTX and ensure the effectiveness of chemotherapy. The combined intervention was then tested at the cellular level using human and murine colon cancer cell lines. The results of CCK-8 analysis, colony formation and live-dead cell staining experiments indicated that for the cells treated with SCP NPs, the introduction of laser and ultrasound greatly reduced the cell survival rate and killed a large number of cells. The laser power was 1 W/cm^2^ and the maximum temperature did not exceed 43°C. The high temperature maintenance time was also within 3 min. The ultrasound power is 0.5 W/cm^2^. With only chemotherapy or PTT, many cells still survived under such low intervention intensity, showing a significant statistical difference. The ROS levels in cells and tumor tissues showed that the SCP NPs group fully exerted the effect of IR-780 on PDT and SDT. The scenario is due to the fact that SCP NPs carried oxygen into the tumor and effectively relieved tumor hypoxia. The expression of HIF-1α was significantly reduced. It is worth noting that ultrasound treatment can strengthen the ability of SCP NPs to relieve hypoxia. *In vivo* delivery results showed that SCP NPs can effectively deliver therapeutic molecules to the tumor area in both subcutaneous and orthotopic tumor models. The anti-tumor effect *in vivo* proved the synergistic effect of SCP NPs. In the tumor model, the temperature of the tumor area did not exceed 43°C after laser irradiation, and the maintenance time was not long. Hence, SCP NPs would not easily cause low-temperature burns to surrounding tissues. Even so, the tumor volume and tumor weight of the SCP NPs combined intervention group were significantly different from those of other groups. After 3 weeks of intervention, all mice in this group had no visible tumors. Two weeks later, 4 mice in this group had no visible tumors. The mouse tumor area was completely healed, and only 2 mice had recurrence. The overall cure rate was 67%. More importantly, compared with the gradual weight loss of mice in other groups, the weight of mice in the SCP NPs group was also relatively stable and began to rise in the later stages. These results fully proved the excellence of SCP NPs in terms of treatment efficacy. In comparison, the nano-sized drug delivery systems in other reports of the same dosage or intervention methods did not have such excellent results as in this study (Liang S. et al., 2020; Shi et al., 2022; Li et al., 2023).

## 5 Conclusion

In this study, a nanosystem with multiple triggered releases and synergistic effects of chemotherapy, PT, and SDT was designed to address the existing problems in colon cancer treatment. The prepared SCP NPs fully met the design expectations. They can enter the body circulation through intravenous injection and have excellent *in vitro* and *in vivo* stability. The release of SCP NPs accumulated in the tumor can be triggered by ultrasound, low pH, and photothermal, prompting the PDA shell to be broken and released IR-780 and MTX. At the same time, the high oxygen-carrying effect of PFH can also effectively alleviate tumor hypoxia, laying the foundation for subsequent treatment. Then, under the treatment of ultrasound and laser irradiation, the effects of PT, and SDT were simultaneously exerted to treat tumor cells. Finally, MTX was used to kill the remaining tumor cells and empower the synergistic effect. All steps have been verified by relevant experiments. This study can provide experimental basis for the development of new treatment methods for colon cancer.

## Data Availability

The raw data supporting the conclusions of this article will be made available by the authors, without undue reservation.
