# Supplementary material for: Multi-modal triggered-release sonodynamic/chemo/phototherapy synergistic nanocarriers for the treatment of colon cancer
Source: Front Bioeng Biotechnol. 2024 Jul 22;12:1439883. doi: 10.3389/fbioe.2024.1439883 (PMC11298370; doi:10.3389/fbioe.2024.1439883)
Supplement: Supplementary file 1 [file Image1.PDF]

Supplementary Material

Supplementary Figures

1.1 Supplementary Figures

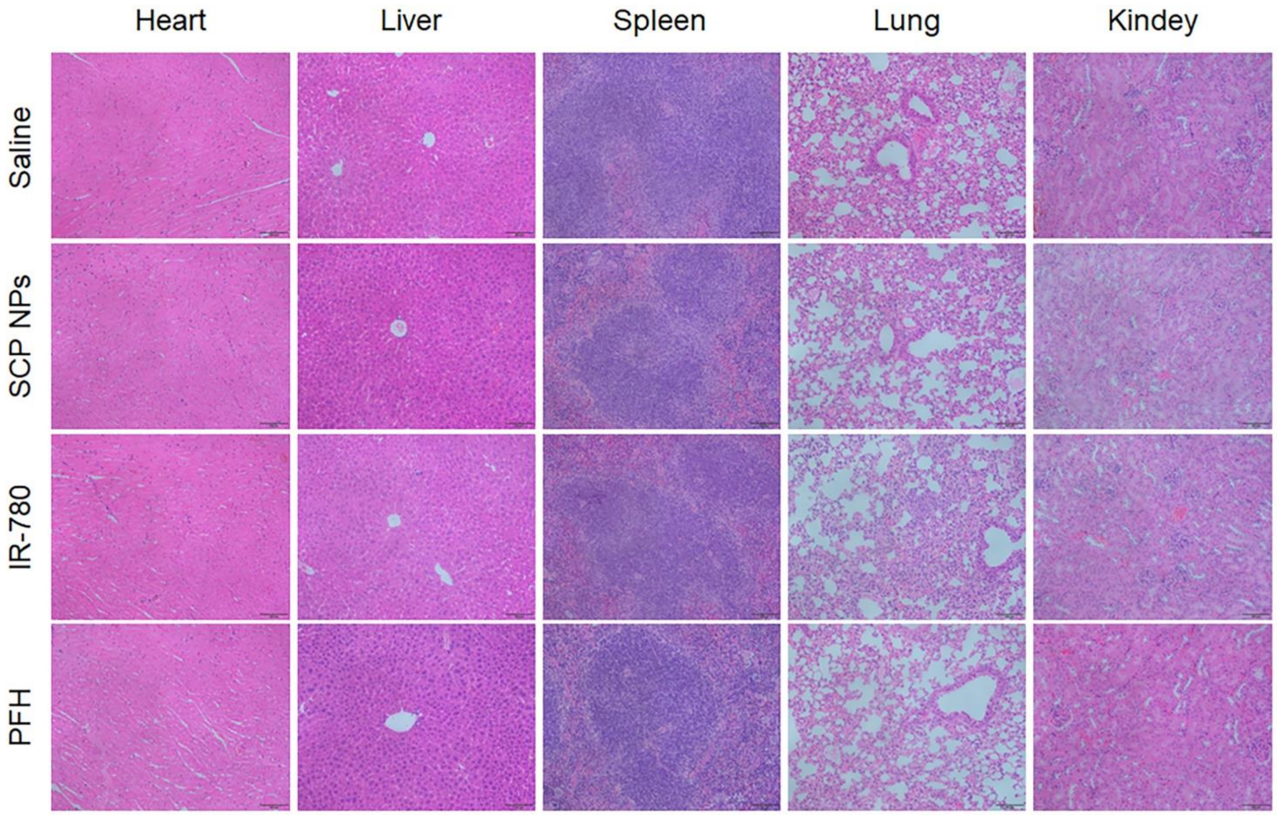

Supplementary Figure S1. The HE staining results of major organ tissues of mice in each group in the second week of acute toxicity test
